# *MePAL6* regulates lignin accumulation to shape cassava resistance against two-spotted spider mite

**DOI:** 10.3389/fpls.2022.1067695

**Published:** 2023-01-06

**Authors:** Xiaowen Yao, Xiao Liang, Qing Chen, Ying Liu, Chunling Wu, Mufeng Wu, Jun Shui, Yang Qiao, Yao Zhang, Yue Geng

**Affiliations:** ^1^ Environment and Plant Protection Institute, Chinese Academy of Tropical Agricultural Sciences/Key Laboratory of Integrated Pest Management on Tropical Crops, Ministry of Agriculture and Rural Affairs, Haikou, Hainan, China; ^2^ Sanya Research Academy, Chinese Academy of Tropical Agriculture Science/Hainan Key Laboratory for Biosafety Monitoring and Molecular Breeding in Off-Season Reproduction Regions, Sanya, Hainan, China

**Keywords:** cassava, two-spotted spider mite, PAL gene family, lignin biosynthesis, VIGS, mite-resistance

## Abstract

**Introduction:**

The two-spotted spider mite (TSSM) is a devastating pest of cassava production in China. Lignin is considered as an important defensive barrier against pests and diseases, several genes participate in lignin biosynthesis, however, how these genes modulate lignin accumulation in cassava and shape TSSM-resistance is largely unknown.

**Methods:**

To fill this knowledge gap, while under TSSM infestation, the cassava lignin biosynthesis related genes were subjected to expression pattern analysis followed by family identification, and genes with significant induction were used for further function exploration.

**Results:**

Most genes involved in lignin biosynthesis were up-regulated when the mite-resistant cassava cultivars were infested by TSSM, noticeably, the MePAL gene presented the most vigorous induction among these genes. Therefore, we paid more attention to dissect the function of MePAL gene during cassava-TSSM interaction. Gene family identification showed that there are 6 MePAL members identified in cassava genome, further phylogenetic analysis, gene duplication, cis-elements and conserved motif prediction speculated that these genes may probably contribute to biotic stress responses in cassava. The transcription profile of the 6 MePAL genes in TSSM-resistant cassava cultivar SC9 indicated a universal up-regulation pattern. To further elucidate the potential correlation between MePAL expression and TSSM-resistance, the most strongly induced gene MePAL6 were silenced using virus-induced gene silencing (VIGS) assay, we found that silencing of MePAL6 in SC9 not only simultaneously suppressed the expression of other lignin biosynthesis genes such as 4-coumarate--CoA ligase (4CL), hydroxycinnamoyltransferase (HCT) and cinnamoyl-CoA reductase (CCR), but also resulted in decrease of lignin content. Ultimately, the suppression of MePAL6 in SC9 can lead to significant deterioration of TSSM-resistance.

**Discussion:**

This study accurately identified MePAL6 as critical genes in conferring cassava resistance to TSSM, which could be considered as promising marker gene for evaluating cassava resistance to insect pest.

## 1 Introduction

Cassava (*Manihot esculenta* Crantz) is a tuber crop that is widely cultivated in more than 100 countries (Parmar et al., 2017). Due to its high environmental adaptability, cassava is one of the most resilient crops as food, feed and biomass energy that served more than 800 millions of people all over the world (Wu et al., 2022; Amelework and Bairu, 2022). By 2017, the global cassava production will increase to 322 million tons and the planting area will be 26 million hectares (FAOSTAT, 2019). China’s total cassava import in 2017 stood at $82.5million and ranked number one in the world (Otekunrin and Sawicka, 2019).

The two-spotted spider mite (TSSM; *Tetranychus urticae*; Acari: Tetranychidae) is the one of the most polyphagous insect pest and poses serious threat to many crops including cassava (Migeon et al., 2010). In China, yield losses that caused by TSSM usually ranged from 50 to 70% (Ngongo et al., 2022). To date, pesticide application is still the routine approach to control TSSM. However, the dense canopy of the cassava plant make the pesticide difficult to target, moreover, the inappropriate pesticides application may also lead to resistance problem (Van Leeuwen et al., 2008). Biological control is also an alternative strategy, and considerable efforts had been made to control cassava mites in the past decades (Herren and Neuenschwander, 1991; Gutierrez et al., 1988; Onzo et al., 2005). Nevertheless, the unstable control efficiency and relatively high cost at the startup, as well as the weak compatibility with other control method (i.e., chemical control) hinder the extension of this technology (Collier et al., 2007). Utilization of pest-resistant plant provides an economical, effective, environmental-friendly strategy for cassava pests management. There are several studies aiming to screen or identify mite-resistant cassava varieties (Bellotti et al., 1999; Bellotti et al., 2012; Parsa et al., 2015). In addition, mapping mite-resistance genes in cassava genome also make good progress (Nzuki et al., 2017; Ezenwaka et al., 2018; Ezenwaka et al., 2020). However, the control methods mentioned above were basically focused on cassava green mite (the *Mononychellus* spp.), in contrary, studies on TSSM were relatively limited. To demonstrate why certain plant was resistant to insect pests could promote the development of control strategy, however, the molecular-based mechanism of cassava resistance to TSSM were largely unknown.

Lignin is the one of the most abundant biopolymer in plant (Jeffrey, 2014). As a complex phenolic polymer, it can enhance both the rigidity and thickness of the cell wall (Vanholme et al., 2008). Thus, lignin is recognized as an important defensive barrier against pests and diseases. Lignin was reported to be associated with the maize resistance to second-generation of European corn borer (*Ostrinia nubilalis*) (Buendgen et al., 1990). Lignin accumulation in the root was regulated by the ethylene- and phenylpropanoid-dependent pathway, which may induce tomato and *Arabidopsis* resistance to the root-knot nematode penetration (Fujimoto et al., 2015). In addition, local lignin deposition as well as salic acid biosynthesis was modulated by *Walls Are Thin* (*WAT)* genes, which will participate in cotton resistance against *Verticillium dahlia* (Tang et al., 2019). Apart from lignin, several intermediate compounds during lignin biosynthesis also possess the pest resistance capacity. For examples, the resistant cotton varieties presented high content of ferulic acid, which significantly delayed the larval weight gain and increased the mortality of cotton bollworm (*Helicoverpa armigera*) (Mao et al., 2007), moreover, targeted metabolome analysis showed higher elevation of trans-cinnamic acid, caffeine and ferulic acid after the *Malus sieversii* resistant strains were infested by *Agrilus mali* (Mei et al., 2020).

The lignin biosynthesis pathway, which was embedded in the phenylpropane pathway, was regulated by a battery of specific genes. The lignin biosynthesis pathways were well illustrated in several plant species including *Arabidopsis thaliana* (Vanholme et al., 2012), tobacco (Shi et al., 2022), poplar (Shen et al., 2021), tomato (Zhang et al., 2017), pepper (Li et al., 2019), and cassava (Ding et al., 2020). Phenylalanine ammonia-lyase (PAL, E.C. 4.3.1.5) is the first key enzyme in the biosynthesis of lignin and other various phenolic compounds like polyphenols and phenolic acids (Ritter and Schulz, 2004). The expression of *PAL* in plant usually associates with environmental stress, abiotic and biotic factors such as pathogen infection (Joshi et al., 2022), mechanical damage (Liu, M. M. et al., 2017), ultraviolet radiation (Pluskota et al., 2005), chemical treatment (Song and Ebizuka, 1996) and extreme temperatures (Ratanamarno et al., 2005), may significantly alter the transcription or enzymatic activity of PAL. In addition, *PAL* is a micro gene family with multiple genes that has been identified and extensively studied in various plants (Wu et al., 2017). Studies have shown that different *PAL* genes perform different functions. The *pal1* and *pal2* genes were contributed to anthocyanin pigmentation in *A. thaliana*, and mutants of those two genes produced yellowish seeds and were highly sensitive to UV-B light (Huang et al., 2010). Transfection of *Pyrus bretschneideri* genes *PbPAL1* and *PbPAL2* into *A. thaliana* caused a remarkable elevation of lignin content and thickening of the cell walls of intervascular fibers and xylem cells (Li et al., 2019). Overexpression of *Ipomoea batatas* gene *IbPAL1* conferred chlorogenic acid accumulation in sweet potato leaves, which would stimulate secondary xylem cell expansion in stems, and inhibited storage root formation (Yu et al., 2021). The *McPAL3* gene in noni fruit (*Mofinda citfifolia*) was confirmed to be a key gene involved in the accumulation of scopoletin, as consistent change trend between scopoletin content and total PAL activity were detected while the plant was treated with ethylen (Wang, H. et al., 2021). In addition, several groups of transcription factors i.e., MYB (myeloblastosis) (Xie et al., 2020), bHLH (basic helix-loop-helix) (Du et al., 2018) and WRKY (Wang, Q. et al., 2021 ), are also essential for regulating the lignin biosynthesis pathway.

During plant-herbivorous pest interaction, although the expression patterns of *PAL* genes had been elaborated (Duan et al., 2014; Fujimoto et al., 2015; Veronico et al., 2018), there is lack of robust evidence of their function in pest resistance. Moreover, the *PAL* gene family in cassava has not been identified and genetically characterized yet, so far as we know, the biological functions of *PALs* in cassava are still mysterious. To fill this knowledge gap, we aim to systematically identify the *PAL* gene family in cassava, and their chromosomal locations, collinearity, classification, evolution, and expression patterns were analyzed. Furthermore, the expression patterns of the identified *PAL* family genes were evaluated when cassava plants were under TSSM-infestation, moreover, the most strongly induced genes were used to validate their functions in lignin biosynthesis and TSSM-resistance. This study could excavate crucial gene that participates in shaping cassava resistance to TSSM, which could be used for evaluating cassava resistance to TSSM, and moreover, assist in the molecular breeding of TSSM-resistant cassava.

## 2 Materials and methods

### 2.1 Cassava materials

Three TSSM-resistant cassava cultivars (C1115, Miandian and SC9) and three TSSM-susceptible cassava cultivars (SC205, Bread and BAR900) that were identified in our previous study (Liang et al., 2022) were supplied by the National Cassava Germplasm Nursery of China, Chinese Academy of Tropical Agricultural Sciences (CATAS). Cassava stem of about 20 cm length were vertically planted with nutritive soil (equal quantity of soil, peat and perlite) in the pot and grown in a greenhouse for TSSM-resistance identification. The light/dark photoperiod was set as 14 h/10 h, temperature was maintained at 28 ± 1°C, and relative humidity was kept at 75 ± 5%.

### 2.2 TSSM rearing, infestation and sample collection

TSSM rearing was conducted based on our previously study (Chen Q. et al., 2022). Three mature and healthy cassava leaves with identical growth status from the middle of the 3-month-old plants were selected, and 50 healthy 1-day old female adult mites were infested on abaxial leaves of either resistant or susceptible cassava cultivars, besides, petioles were coated with lanolin to avoid TSSM escape. Leaves without TSSM infestation, 1 day post infestation (dpi) and 4 dpi were sampled (make sure all the mites were eliminated from the sampled leaves). Each treatment repeated three times (Three plants for one treatment, and each plants sampled three leaves).

### 2.3 RNA extraction and qPCR analysis

Total RNA was extracted from 0.1g of leaf sample using a RNAprep Pure Plant plus Kit (Polysaccharides & Polyphenolics-rich, Tiangen, China). RT EasyMix for qPCR (+2-Step gDNA Erase-out, Tolobio, China) was used for first-strand cDNA synthesis. The qPCR reaction system was prepared according to the 2×Q3 SYBR qPCR Master Mix kit (Tolobio, China). The qPCR reactions were performed in 10 μL volume in QuantStudio 6 Flex (Thermo Fisher, America) with three biological replicates as described previous. *PAL*, 4-coumarate–CoA ligase (*4CL*), shikimate O-hydroxycinnamoyltransferase (*HCT*), trans-cinnamate 4-monooxygenase (*C4H*), caffeoylshikimate esterase (*CSE*), caffeoyl-CoA O-methyltransferase (*CCoAOMT*), caffeic acid 3-O-methyltransferase (*COMT*), ferulate-5-hydroxylase (*F5H*), cinnamoyl-CoA reductase (*CCR*) and cinnamyl-alcohol dehydrogenase (*CAD*), Using the Primer5 to design specific primers according to the conservative domain of lignin biosynthesis gene. The qPCR primers were shown in **Supplementary Table S1**, *MeActin* was used as reference gene. The RT-qPCR conditions were: an initial denaturation for 2 min at 95°C, followed by 40 cycles of denaturation at 95°C for 5 s and annealing at 60°C for 30 s, and a final elongation step at 72°C for 60 s. For the melting curve analysis, a dissociation step cycle (65°C for 5 s, and then an increase of 0.5°C every 10 s up to 95°C) was used. The relative gene transcription was calculated based on the comparative 2^-ΔΔCt^ method (Livak and Schmittgen, 2001).

### 2.4 Determination of enzyme activity and lignin content

The activities of lignin biosynthesis pathway enzymes, such as PAL, 4CL, CCR and HCT, and the lignin content were all analyzed using ELISA kits (Shanghai Enzyme-linked Biotechnology Co., Ltd, Shanghai, China) according to the manufacturer’s instructions (Guo et al., 2022). Each treatment repeated three times.

### 2.5 Identification of *MePAL* genes in cassava genome

The whole cassava genome sequence (v.8.0) and annotation were derived from the phytozome database^1^. The Hidden Markov Model profile of Aromatic amino acid lyase (PF00221) was retrieved from Pfam^2^ (Finn et al., 2015). The TBtools program (Chen et al., 2020) was used to search for Aromatic amino acid lyase in cassava genome, and then the predicted putative domains were further confirmed by the NCBI Conserved Domain Database^3^. The MW (molecular weight) and pI (theoretical isoelectric point) of these identified *MePAL* genes were predicted by ExPASy^4^ (Artimo et al., 2012). Finally, each identified *MePAL* gene was mapped on the chromosome, and all the *MePAL* genes were named consecutively (start with *MePAL1*) according to the orders of the chromosomes.

### 2.6 Chromosomal mapping, gene structure and conserved motif analysis

The chromosomal mapping, gene structures and conserved motif analysis were visualized using the Tbtools. The locations of these *MePAL* genes were determined by querying the cassava genome. In addition, the conserved motifs among all *MePAL* genes were identified *via* online software MEME^5^ (Bailey et al., 2015).

### 2.7 Cis-acting regulatory element analysis

The 2,000 bp sequences from upstream of the transcription start site ATG were extracted as the putative promoter region, and then PlantCare^6^ (Higo et al., 1999) database were used to screen and identify the cis-acting regulatory elements. The components predicted by the PlantCare online tool were first screened, and the unannotated components were eliminated while the annotated component were sorted out, after that, the components with consistent functions shared with the identical function annotations, and element visualization was conducted by using Tbtools.

### 2.8 Phylogenetic analysis, gene duplication, multiple alignments and synteny analysis

Phylogenetic analysis of *PAL* genes from four plant species, i.e., *M. esculenta*, *Ricinus communis*, *Hevea brasiliensis* and *A. thaliana*, were conducted by MEGA (version 11.0). A phylogenetic tree was constructed with the neighbor-joining method and the parameters were Jones-Taylor-Thornton model, pairwise deletion, and 1,000 bootstrap replicates. Circos program (Krzywinski et al., 2009) was used to illustrate the relationships of duplicated genes. The collinearity analysis of *PAL* genes between *M. esculenta* and other three species were detected by One Step MCScanX. KaKs was used to calculate the nonsynonymous replacement rate (Ka) and synonymous replacement rate (Ks), where Ka/Ks1 means positive selection, Ka/Ks1 means purifying selection and Ka/Ks = 1 means neutral evolution.

### 2.9 Virus-induced gene silencing (VIGS) in cassava followed by TSSM infestation

The TSSM-resistant cassava cultivar SC9 was used as the transfected plant. For vector construction, 300 bp *MePAL6* and 300 bp *MeCHI* (cassava chelatase subunit I gene that accounts for chlorophyll synthesis) DNA fragments were cloned and linked into CsCMV-NC vector as described by Tuo et al. (2021), and SGN-VIGS^7^ (Fernandez-Pozo et al., 2015) online tool was used to select the targeted regions. After silencing of *MeCHI*, the leaves were supposed to be chlorosis and whitening, which is used as a positive control (Tuo et al., 2021). CsCMV-NC (empty plasmid) was used as a negative control to detect the effect of no-load on cassava plants. All the plasmids (recombinant or empty plasmids) were transformed into Agrobacterium-competent cell GV3101 and cultured in a constant temperature incubator at 28°C for 2-3 days. Once the positive control present obvious chlorosis and whitening symptoms, the leaves of *MePAL6*-silenced lines were sampled and the silenced efficiency was measured.

### 2.10 Performance of *MePAL6*-silenced cassava lines against TSSM infestation

After confirming the silencing of the *MePAL6* gene, mites were inoculated on the *MePAL6*-silenced and negative control plants, according to the method described above (Section 2.2). The TSSM infestation symptoms were recorded on 0, 1 and 4 dpi, respectively. In addition, the TSSM-resistance levels of each tested plant were evaluated (conducted on 4 dpi) according to the mite damage index method (**Supplementary Figure S1**). Furthermore, the expression of other *MePAL* members, the downstream lignin biosynthesis genes and the lignin content in *MePAL6*-silenced lines were also detected. The primer information was listed in **Supplementary Table S1**.

### 2.11 Statistical analysis

All data analyses were performed using the SPSS (Version 26.0), and statistical analysis was conducted using one-way analysis of variance (ANOVA) with Tukey’s honestly significant difference (HSD) multiple comparison test. Pearson’s correlation test was used to calculate the correlation coefficients among mite damage indexes, transcription of genes, enzyme activities and lignin contents at 4 dpi. Significant and highly-significant difference were considered if P-values were < 0.05 and < 0.01, respectively.

## 3 Results

### 3.1 Influence on lignin biosynthesis pathway while cassava was infested by TSSM

**Figure 1:**
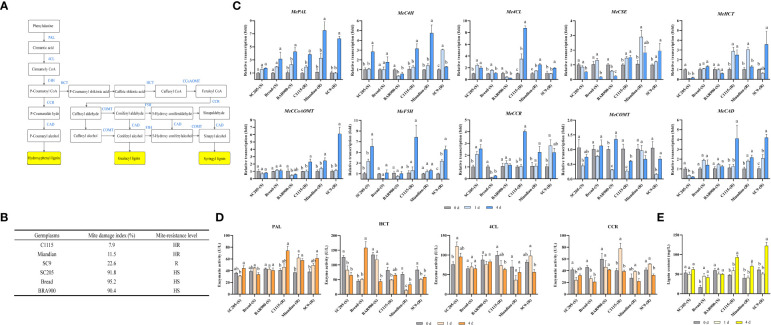
Influence on lignin biosynthesis pathway while TSSM-resistant and TSSM-susceptible cassava cultivars were infested by TSSM. **(A)** The potential schematic diagram of lignin biosynthesis pathway in cassava. The biosynthesis genes, the intermediate products and the final different forms of lignin products were marked with blue letters, white frames and yellow frames, respectively; **(B)** The mite damage indexes and the corresponding resistance levels of the tested cassava cultivars; **(C)** Changes in transcription of ten lignin biosynthesis genes; **(D)** Changes in activity of four enzymes involved in lignin biosynthesis pathway; **(E)** Changes in lignin content. Different letters above standard error bars indicate significant differences based on ANOVA followed by Tukey’s HSD multiple comparison test (*p* < 0.05) within the same time point.

After TSSM infestation, the transcription of lignin biosynthesis pathway genes (**Figure 1A**) in TSSM-resistant and TSSM-susceptible cassava cultivars presented distinct patterns (The mite damage indexes which were used to interpret the resistance level of each tested cultivars was presented in **Figure 1B**). The transcriptions of *MePAL*, *Me4CL*, *MeCCoAOMT*, *MeCCR* and *MeCAD* in three TSSM-resistant cultivars (C1115, Miandian and SC9) were significantly increased over time (*P* < 0.05), while the transcriptions of *MeC4H*, *MeHCT*, *MeCSE* and *MeF5H* in certain TSSM-resistant cultivars (SC9 and Miandian) were first increased (1 dpi) and then decreased (4 dpi). In addition, the gene transcription in the three TSSM-susceptible cassava cultivars (SC205, Bread and BRA900) showed inconsistent change pattern with *MePAL* as an exception, in which the transcriptions were continuously increased (1.21- to 2.31-fold relative to those before infestation), in comparison, the transcriptions of *MePAL* in TSSM-resistant cultivars were much higher after TSSM infestation (3.83- to 6.49-fold relative to those before infestation) (**Figure 1C**).

Enzyme activities were analyzed to examine whether the transcription and post- transcription of the lignin pathway genes showed identical change trend. The four genes, i.e., *MePAL*, *Me4CL*, *MeCCR* and *MeHCT* that showed stable and significant elevation of transcription were selected. The result illustrated that only PAL demonstrate significantly higher enzyme activity in the TSSM-resistant cultivars compared with the susceptible ones, which was consistent with the transcription levels. However, the rest of three genes did not show consistency between gene transcription and enzyme activity, besides, the activities of 4CL, CCR and HCT in TSSM-resistant cultivars were not necessarily higher than those of susceptible ones (**Figure 1D**).

After TSSM infestation, the elevation of lignin contents in C1115-(R), Miandian-(R) and SC9-(R) posed a hysteretic manner, as compared with those before infestation, the lignin contents remained unchanged on 1 dpi but significantly increased on 4 dpi (*P* < 0.05). It is noteworthy that the lignin contents in SC205-(S) and BRA900-(S) did not showed statistically difference (*P* < 0.05), although Bread-(S) possessed constitutively low lignin content before mite infestation, TSSM infestation would significantly trigger the lignin accumulation. In comparison, during TSSM-cassava interaction, especially on long term exposure (4 dpi), the lignin contents in all the resistant cultivars were significantly higher than the susceptible ones (**Figure 1E**).

Correlation analysis was conducted among mite damage indexes, transcription of genes, enzyme activities and lignin contents. It was noticeable that the gene transcriptions and lignin contents were moderately- and highly-correlated with mite damage, while the enzyme activities only showed pretty low correlation with mite damage (**Table 1**).

**Table 1:** Correlation among mite damage indexes, transcription of genes, enzyme activities and lignin contents.

	Transcription level	Enzyme activities	Lignin content	Mite damage index
Transcription level	1	-0.09^NS^	0.399 ^NS^	-0.541^*^
Enzyme activities		1	-0.86 ^NS^	0.149 ^NS^
Lignin content			1	-0.719^**^
Mite damage index				1

NS indicates non-significance, one asterisk and two asterisks indicate significant (P < 0.05) and highly-significant difference (P < 0.01), respectively.

### 3.2 Identification of *MePAL* gene family in cassava

**Figure 2:**
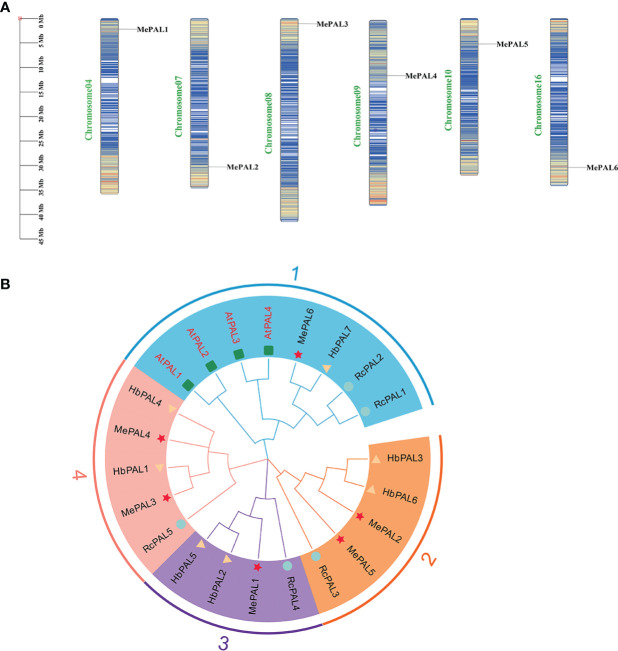
The chromosomal locations and phylogeny of *MePALs*. **(A)** Chromosomal locations of the six *MePAL* genes. The chromosomes were presented as narrow rectangles, and color bars within the rectangles denoted the *M. esculenta* chromosome density. Scale bars on the left indicated the chromosome lengths (Mb); **(B)** Phylogenetic tree of the 6 MePAL proteins. The tree was constructed by using MEGA X based on the full-length amino acid sequences from *M. esculenta* (Me) (marked with stars), *R. communis* (Rc) (marked with circles), *H brasiliensis* (Hb) (marked with triangles) and *A thaliana* (At) (marked with boxes). All nodes had significant bootstrap support based on 1,000 replicates. The tree was constructed with cut-off value of 50%. Genes that distributed in the same clusters were shadowed with different colors.

A total of 6 MePAL proteins were characterized from cassava and named them from MePAL1 to MePAL6. These 6 MePALs all contained the aromatic amino acid lyase domain based on Pfam analysis. MePAL protein lengths were ranged from 703 (MePAL2) to 790 (MePAL4) amino acids, MW were from 64.47 to 86.14 kDa, and pI were from 5.93 to 6.31. In addition, subcellular localization of MePALs was predicted by WoLF PSORT10^8^. Only MePAL1 were localized on the endoplasmic reticulum, while the remaining MePALs were localized on the chloroplast. More detailed information including *MePAL* gene annotation, gene accession, chromosome locus, protein length, MW, pI of all identified MePAL were shown in **Supplementary Table S2**. To further investigate the chromosomal distribution of the *MePAL* genes, the DNA sequence of each *MePAL* was obtained using Blastn in cassava genome database, and these 6 *MePAL* genes were mapped on 6 chromosomes (**Figure 2A**).

The amino acid sequences of PALs deriving from three Euphorbiaceae plant species, i.e., *M. esculenta*, *R. communis*, *H. brasiliensis* together with a model plant *A. thaliana* were used to construct a phylogenetic tree by using neighbor-joining method. All the *PALs* from different plant species were clustered into 4 groups. Group 1 contained the largest *PAL* gene members (8 *PALs*), followed by Group 2 and Group 4 with 5 *PALs*. The 6 *MePALs* were evenly distributed in each group which contained at least one *PAL* gene (**Figure 2B**), In addition, it is noteworthy that *MePAL6* were clustered with all the 4 *AtPAL* genes in Group 1, indicating that the evolution conservation between MePAL6 and *AtPALs*, moreover, as the *PAL* gene function in all the three Euphorbiaceae plants were not identified so far, while the function of *AtPALs* in dealing with environmental stress had been well-studied (Wanner et al., 1995), based on the above information, we assumed that specific gene in cassava (i.e., *MePAL6*) which was clustered together with the *AtPALs* maybe responsible for abiotic or biotic stresses.

### 3.3 *MePAL* gene structures, conserved motifs and cis-acting regulatory element analysis

**Figure 3:**
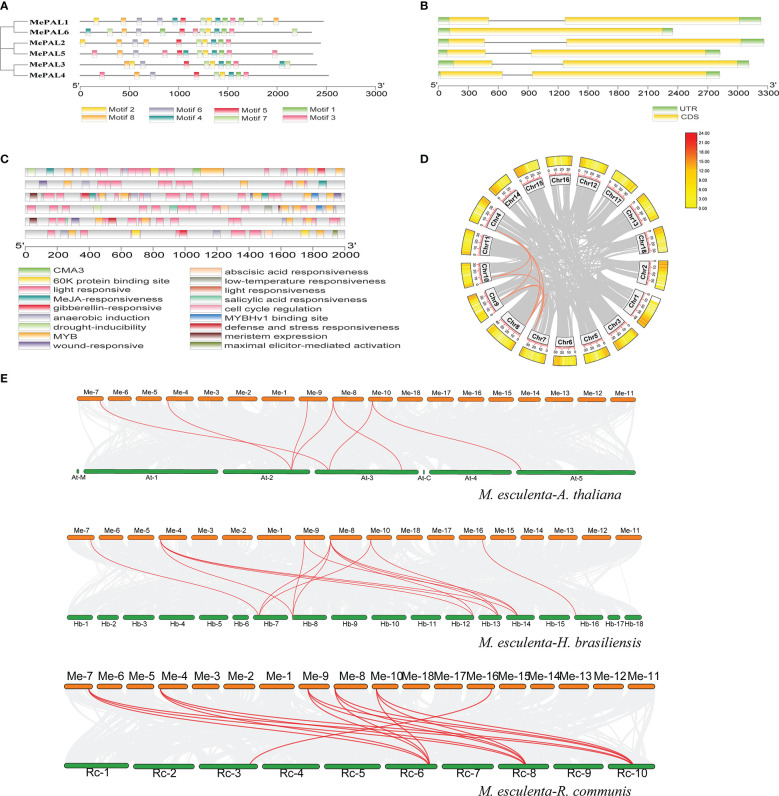
Conserved motifs, gene structures, Cis-acting element and gene duplicate of the *MePAL* genes. **(A)** MEME analysis revealed the conserved motifs of the MePAL proteins. The colored boxes at the bottom denoted 8 motifs; **(B)** Structures of the six *MePAL* genes. The yellow boxes, black lines, and green boxes represented exon, intron, and UTR (untranslated region), respectively; **(C)** Prediction of cis-acting elements and visualization with Tbtools, the colored boxes at the bottom indicated the predicted elements; **(D)** Circos diagram of the MePAL duplication pairs in *M. esculenta*. The outer boxes indicated the gene density of each chromosomes, and the interior orange and grey curves indicated the collinearity relationships among *MePAL* genes and all the genes in the chromosomes, respectively; **(E)** Collinearity analysis between *M. esculenta* and *A thaliana, M. esculenta* and *H brasiliensis, M. esculenta* and *R. communis*. The interior red curves indicated the collinearity relationships of *MePAL* genes between two plant species, while the grey curves indicated the collinearity relationships of all the genes in the chromosomes between two plant species, respectively.

The exon/intron organization and conserved motifs of all *MePAL* genes were analyzed. Eight conserved motifs with multiple repeats were identified among the 6 MePAL proteins, besides, the motifs number was ranged from 9 (*MePAL4*) to 14 (*MePAL1*, *MePAL5*), although *MePAL4* contains only 9 motifs, it possessed the longest amino acid sequence among all MePALs (**Figure 3A**), in addition, sequence alignment indicated that the similarity of the 6 MePAL proteins was 77.01% (**Supplementary Figure S2**). All *MePAL* genes had 2 exons and 2 introns, except for *MePAL6*, which had only one intron (**Figure 3B**).

PlantCARE database was used to characterize the cis-acting regulatory elements (CAREs) of each *MePAL* gene. Light responsiveness and MYB elements were presented in all 6 *MePALs*. Furthermore, CAREs related to hormone responses were also identified, such as Methyl jasmonate (MeJA)-responsiveness, abscisic acid responsiveness and gibberellin-responsive element. Interestingly, the CAREs of some *MePALs* also contained elements related to biotic and abiotic responses, including wound-responsive element, anaerobic induction, MYB binding site involved drought-inducibility, defense and stress responsiveness. In summary, diverse hormone and environmental factors might affect the expression of *MePAL* genes (**Figure 3C** and **Supplementary Table S3**).

To reveal the expansion mechanism of the *MePAL* genes, CDS of all *MePAL* genes were subjected to Blastn within the cassava genome. Totally, 8 pairs (5 *MePAL* genes) of segmental duplications as well as 8 pairs of fragment duplications (*MePAL1/MePAL2, MePAL1/MePAL3, MePAL1/MePAL4, MePAL1/MePAL5, MePAL2/MePAL3, MePAL2/MePAL4, MePAL2/MePAL5* and *MePAL3/MePAL4*) were identified (**Figure 3D**). In addition, the Ka/Ks values of all gene pairs were less than 1.0, which indicated that these genes evolved under purification selection (**Supplementary Table S4**). In addition, to detect the synteny of *PAL* genes, a collinearity analysis between *M. esculenta* and other plant species using TBtools were performed. Finally, 7 paired collinearity relationships between 5 *MePAL* and 4 *AtPAL* genes were established, 15 paired collinearity relationships between 6 *MePAL* and 6 *HbPAL* genes, and 16 paired collinearity relationships between 6 *MePAL* and 4 *RcPAL* genes (**Figure 3E**).

### 3.4 The transcription patterns of the 6 *MePAL* genes when SC9-(R) underwent TSSM infestation

**Figure 4:**
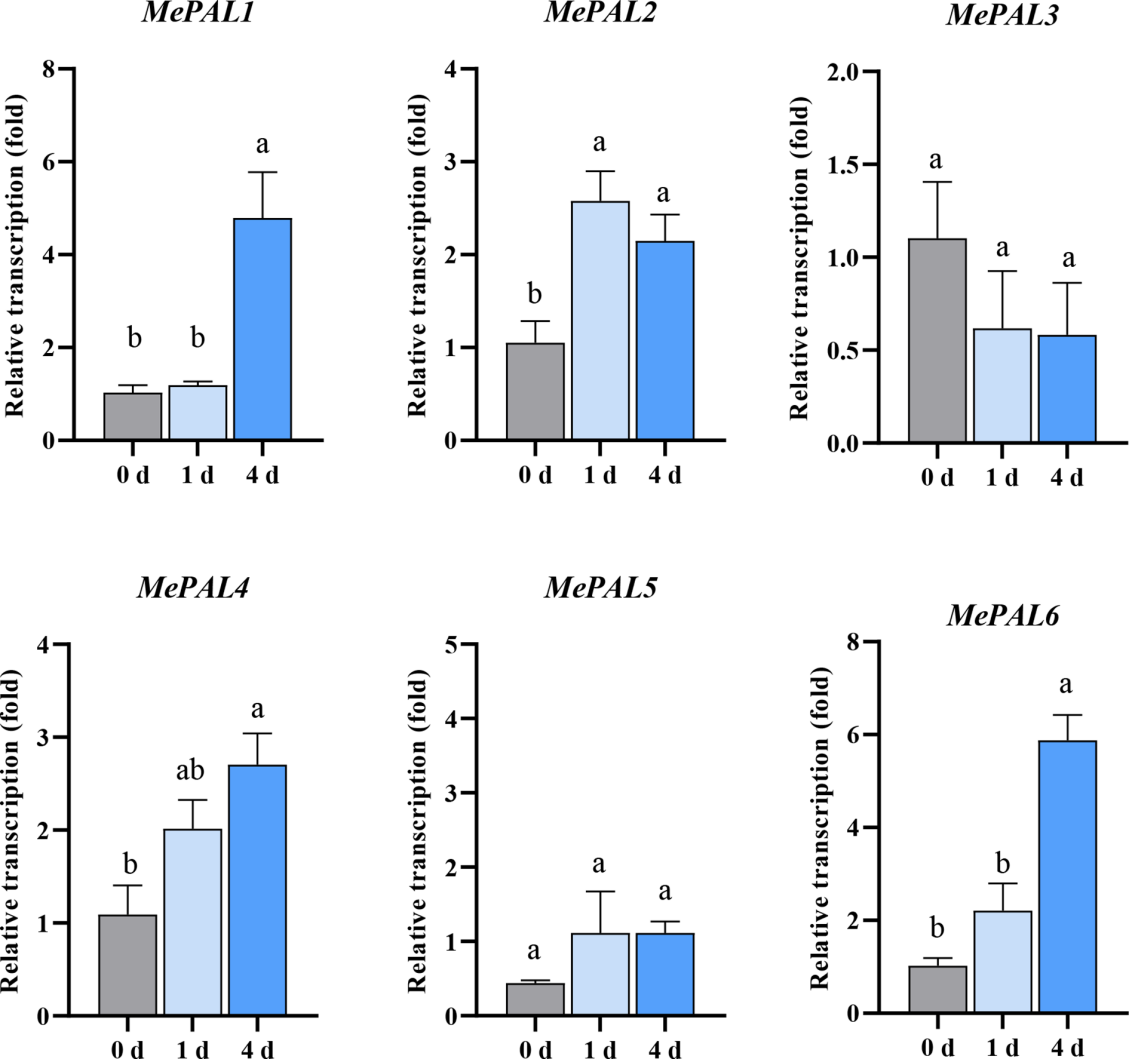
Transcription level of six *MePAL* genes in SC9-(R) after TSSM infestation. Different letters above standard error bars indicate significant differences based on ANOVA followed by Tukey’s HSD multiple comparison test (*p* < 0.05) within the same time point.

In order to delicately speculate the transcription patterns of the 6 identified *MePAL* genes, primers were designed mapping to the specific regions of each gene (**Supplementary Table S1**). After TSSM infestation., the transcriptions of most *MePAL* genes (*MePAL1, MePAL2, MePAL4* and *MePAL6*) significantly increased over time, while the transcription of *MePAL3* significantly decreased, and the transcription of *MePAL5* did not show statistically difference (*P* < 0.05) (**Figure 4**). In particular, the transcription of *MePAL6* increased by approximately 6-fold (at 4 dpi), which was the most strongly induced gene (**Figure 4**). Take into account the vigorous induction as well as the function similarity with model plant (Section 3.2), the *MePAL6* gene, was finally selected to clarify the potential function associated with TSSM-resistance in cassava.

### 3.5 Effects of *MePAL6* silencing on lignin biosynthesis and performance on TSSM resistance

**Figure 5:**
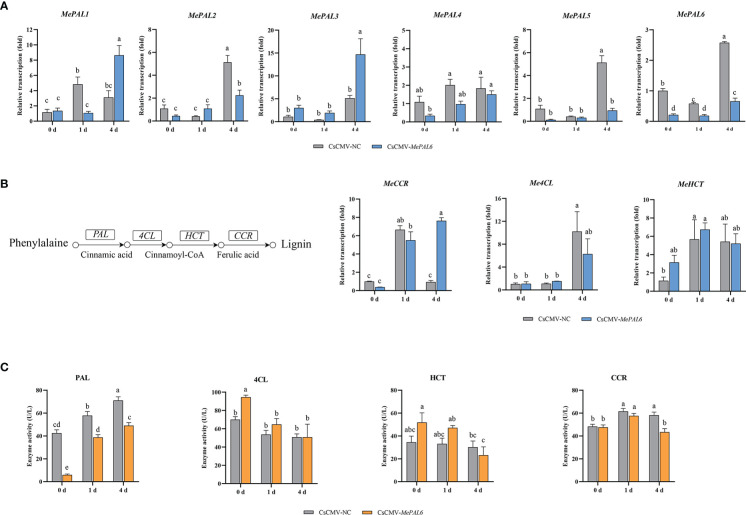
Effects of *MePAL6* silencing on lignin biosynthesis pathway and TSSM-resistance performance of cassava. **(A)** Transcription changes of six *MePAL* genes (*MePAL1*-*MePAL6*) in *MePAL6*-silenced cassava lines while under TSSM infestation; **(B)** Transcription changes of downstream lignin biosynthesis genes (*Me4CL*, *MeCCR* and *MeHCT*) in *MePAL6*-silenced cassava lines while under TSSM infestation, the simplified lignin biosynthesis pathway was presented on the left, and the investigated downstream genes were labeled; **(C)** Enzyme activity changes of PAL, 4CL, CCR and HCT in *MePAL6*-silenced cassava lines while under TSSM infestation. Different letters above standard error bars indicate significant differences based on ANOVA followed by Tukey’s HSD multiple comparison test (*p* < 0.05) within the same time point.

To study the roles of *MePAL* genes in lignin biosynthesis and TSSM-resistance, a *MePAL6*-silenced line CsCMV-*MePAL6* (treatment) was constructed by using VIGS. The chlorosis and whitening phenotype of CsCMV-*MeCHI* line (positive control) indicated the effectiveness of viral inoculation on silenced plant (**Supplementary Figure S3**). In addition, the transcription of *MePAL6* in CsCMV-*MePAL6* were drastically reduced compared with negative control CsCMV-NC (**Figure 5A**) (Silenced efficiency was approximately 78.35%). Furthermore, as the VIGS target region of *MePAL6* showed high identity to the rest of 5 *MePAL* genes, thus, their transcriptions were also investigated. qPCR analysis showed that *MePAL2* and *MePAL5* were also suffered transcription depression, while the transcription of *MePAL1*, *MePAL3* and *MePAL4* were not suppressed but were increased at certain timepoints after TSSM infestation (**Figure 5A**).

Influence on the expression of downstream genes involved in lignin biosynthesis pathway were also examined. The transcription of *Me4CL* and *MeCCR* in the CsCMV-*MePAL6* line significantly increased on 4 dpi, nevertheless, it was still significantly lower than that of negative control CsCMV-NC. In addition, *MeHCT* was also induced after TSSM-infestation, but there was no significant difference between CsCMV-*MePAL6* line and CsCMV-NC (**Figure 5B**). Moreover, enzyme activities were analyzed to examine whether the transcription and post-transcription of the downstream genes showed identical change trend. Results indicated that compared with control, the PAL activity in *MePAL6-*silenced line was significantly decreased (*P* < 0.05), nevertheless, it still can be induced under TSSM-infestation (**Figure 5C**). By contrast, the activities of 4CL, CCR and HCT between control and *MePAL6-*silenced line basically did not show significant differences (*P* < 0.05) (**Figure 5C**).

**Figure 6:**
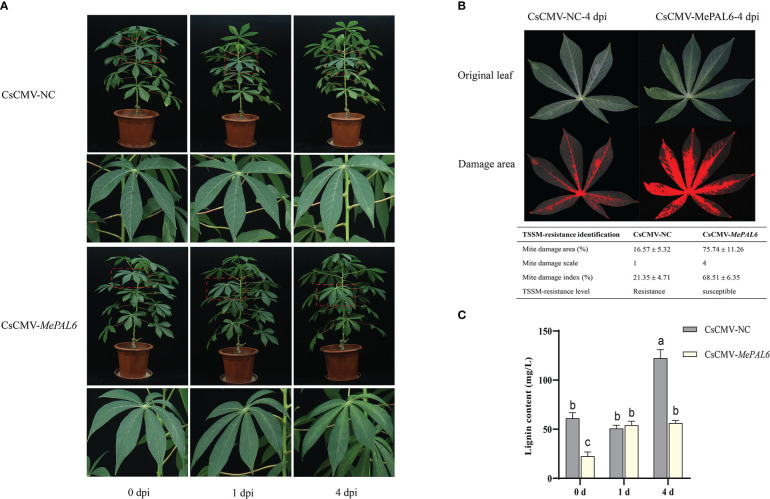
Performance of *MePAL6* silencing lines against TSSM infestation. **(A)** The TSSM infestation symptom of *MePAL6* silencing lines and negative controls. The “zoom in” areas of plants after mite infestation on 0, 1, 4 days were indicated by red dashed boxes. **(B)** Identification of mite damage index after 4 dpi in *MePAL6* silencing lines and negative controls. **(C)** Changes in lignin content in *MePAL6*-silenced cassava lines while under TSSM infestation. Different letters above standard error bars indicate significant differences based on ANOVA followed by Tukey’s HSD multiple comparison test (*p* < 0.05) within the same time point.

Distinct TSSM infestation symptoms were observed between *MePAL6-*silenced line and control. During short-term exposure to TSSM, there were only very slight white and yellow spots on both two lines without difference in symptom. However, on 4 dpi, the *MePAL6-*silenced line suffered serious TSSM infestation symptoms, where dense TSSM damage spots covering the whole leaf, by comparison, there was no symptom deterioration in the control plant (**Figure 6A**). Moreover, the mite damage index of *MePAL6-*silenced line was 68.51% (the TSSM-resistant level was identified as ‘susceptible’), which was higher than that in CsCMV-NC (21.35%, the TSSM-resistant level was identified as ‘resistance’), indicating the TSSM-resistance level of SC9 was shift from resistance to susceptible, after silencing of lignin biosynthesis gene *MePAL6* (**Figure 6B**). In addition, before TSSM infestation, the lignin content in *MePAL6-*silenced line was significantly lower than that in control, suggesting silencing of *MePAL* gene would decrease the lignin production. Moreover, after TSSM infestation, the lignin content in both two cassava lines significantly increased, but the CsCMV-NC presented higher lignin accumulation, especially when exposed to TSSM for a long term (4 dpi) (**Figure 6C**). We speculated that the decrease of lignin content might be the reason of TSSM-resistance deterioration.

## 4 Discussion

### 4.1 The lignin biosynthesis pathway was significantly induced in TSSM-resistant cassava cultivars

Lignin and its intermediate products are crucial defensive substances coping with pest infestation. By serving as physical and chemical barrier, effective accumulation of lignin can significantly enhance plant resistance (Lee et al., 2019). A battery of genes participates in lignin biosynthesis (**Figure 1A**), and those genes could be significantly induced during insect pest invasion. The expression levels of the *PAL, 4CL, COMT* and *CAD* genes in the lignin synthesis pathway increased significantly after *Panax notoginseng* was inoculate with fungal (Yang et al., 2022). Transcriptomic analysis depicted that when Chinese chestnut (*Castanea mollissima*) in response to the chestnut gall wasp (*Dryocosmus kuriphilus*) infestation, the majority of genes associated with the lignin biosynthesis pathway, including *PAL, CAD, CCOAOMT, COMT* and *HCT* were significantly upregulated (Zhu et al., 2019), besides, identical genes with similar upregulation was found in the study regarding soybean-bean pyralid larvae interaction (Zeng et al., 2017). Similar to former studies, here we noticed that almost all the lignin biosynthesis pathway genes were significantly induced while cassava plants encountered TSSM-feeding stress, probably imply the universality of lignin-based defense response in different plants.

The pest-resistant and pest-susceptible plants usually display distinct expression pattern of lignin synthesis genes. For instances, the expression level of *PAL* increased rapidly after insect-resistant rice cultivars was infested by small brown planthopper (*Nilaparvata lugens*), and was significantly higher than the susceptible varieties (Duan et al., 2014), in another study conducted by Panatda (Jannoey et al., 2017), higher expression of *C4H* in resistant rice variety than the susceptible one also can be observed. In addition, when infested by root-knot nematode, both resistant and susceptible tomato cultivars showed expression elevation of lignin biosynthesis genes (*PAL*, *C4H*, *HCT* and *F5H*) at early times (2–4 dpi), and the induction was faster and greater in resistant cultivars after infection (Veronico et al., 2018). Similarily, in present study, stronger gene induction on TSSM-resistant cassava cultivars was also observed, which probably indicated the significance of lignin pathway contributing to cassava resistance to TSSM. Furthermore, as the *MePAL* gene demonstrated the most robust induction (in both transcription and enzymatic level) among all the lignin biosynthesis genes, we therefore focused on revealing the potential function of *MePAL* in conferring cassava resistance to TSSM.

### 4.2 Gene family identification indicated the six *MePAL* genes in cassava present conservative function in dealing with environmental stress

PAL is encoded by a small gene family, and different *PAL* genes usually have multiple functions (Shine et al., 2016). For examples, there were 4 *PAL* genes identified in *A. thaliana*, and three of them (*AtPAL1*, *AtPAL2*, and *AtPAL4*) were highly expressed in lignifying cells-rich inflorescent stems, whereas *AtPAL3* was expressed at a very low level, indicating only the former three genes involved in lignin biosynthesis (Raes et al., 2003). There are 14 *PALs* identified in potato (*Solanum tuberosum*), among them *StPAL1, StPAL6, StPAL8, StPAL12*, and *StPAL13* functioned in the stress defense against high temperature and drought, while *StPAL1, StPAL2*, and *StPAL6* participated in chemical defense mechanisms (Mo et al., 2022). In pepper (*Capsicum annuum*) only *CaPAL1* was found to be responsible for defense against microbial pathogens (Kim and Hwang, 2014). Although the genome of cassava had been sequenced, the gene family identification of *PAL* has not been carried out yet, the function of *PAL* in cassava is largely unknown.

In this study, six *MePAL* genes were identified in cassava genome, and these six *MePAL* genes were distributed on six chromosomes, which were located at the top or bottom of chromosomes. The *PAL* gene family is represented as a mini-family in most plants, for examples, there are four genes in *A.thaliana* (Cochrane et al., 2004), five genes in *Populus trichocarpa* (Shi et al., 2013), nine genes in *Oryza sativa* (Gho et al., 2020), fifteen genes in *Vitis vinifera* (Zhao et al., 2021) and seven genes in *Camellia sinensis* (Chen X et al., 2022). The relative MW and pI values of *MePAL* family genes were similar, indicating that the evolutionary conservation of *MePAL* family genes in cassava. Phylogenetic analysis showed that the *MePAL6* gene was clustered with all the 4 *AtPALs*, as different *AtPAL* genes were validated to cope with abiotic or biotic stress such as pathogen infection, trauma, nutrient depletion, ultraviolet radiation, and extreme temperatures (Huang et al., 2010; Joshi et al., 2022), we assumed that *MePAL6* may also possessed similar function in dealing with environmental stress.

Cis-acting elements analysis of the critical genes will help in the elucidating the molecular mechanisms that associated with plant stress responses (Moghadam et al., 2012; Moghadam et al., 2013). This study indicated that considerable hormone response elements (i.e., MeJA-, salicylic acid-, gibberellin- and abscisic acid responsivenesses) as well as numerous stress response elements (i.e., anaerobic induction, MYB binding site involved drought-inducibility, wound-responsive element and low-temperature responsiveness) probably accounted for the induction of *MePALs* when cassava plant suffered TSSM infestation. Valifard et al. (2014) conducted promoter analysis of *PAL* genes from three plants (*Mimulus guttatus*, *Zea mays* and *A. thaliana*), and several common elements were predicted to be responsible for abscisic acid, alicylic acid, anaerobic induction, heat stress responses, light responses, MeJA-responses and wound responses. In agreement with these findings, studies have also shown the existence of TC-rich repeats (Zhao et al., 2012);, CAAT box, G box, CGTCA motif, TCA-element (Jiang et al., 2013) in *PAL* promoter in accordance with stress hormones may dealing with environmental stress. From present and previous studies, we presumably speculated that the cis-acting elements in different plant species shared similar biological function, and the induction of *PAL* gene could be considered as a universal stress response while plant was under abiotic or biotic stress.

### 4.3 Silencing of *MePAL6* reduces lignin content and attenuates cassava resistance to TSSM

Manipulation the gene expression involved in lignin biosynthesis will significantly alter the lignin accumulation. To date, most genes that participate in lignin biosynthesis had been subjected to establish genetic modified plants, of which the lignin content and disposition were further analyzed. For examples, overexpression of *CCR, F5H, CSE, CCoAOMT* will increase the lignin content in *Paspalum dilatatum* (Giordano et al., 2014), *O. sativa* (Takeda et al., 2017), while silenced the *4CL*, *CAD* and *HCT* will decreased the lignin content in *O. sativa* (Liu, H. et al., 2017), *Populus trichocarpa* (Van Acker et al., 2017), *Populus nigra* (Vanholme et al., 2013). In addition, modification the expression of *CCR*, *HCT* and *CAD* can result in reconfiguration of different lignin units, i.e., S-unit, G-units and H-unit. Besides, some antioxidant enzymes, like *POD* (Herrero et al., 2013) *SOD* and *APX* (Shafi et al., 2015), and certain transcription factors such as MYB (Guo et al., 2017), bHLH (Gao et al., 2019) and WRKY (Guillaumie et al., 2009) also can regulate the lignin biosynthesis. Among those genes, *PAL* attracts great concern. In one hand, molecular genetics methods have been used to silence or disrupt *PAL* genes in numerous plants, like *A. thaliana* (Huang et al., 2010), tobacco (Korth et al., 2001), *Scutellaria baicalensis* (Park et al., 2012) and rice (Fang et al., 2013), not surprisingly, lignin modified plants were successfully constructed. In the other hand, delicate investigations were also performed to make out the specific biological function of certain *PAL* gene member (Kim and Hwang, 2014; Van Acker et al., 2017; Mo et al., 2022)

By silencing one of the *PAL* gene members, the *MePAL6*, we got a cassava line with 48% reduction of lignin content. Interestingly, the expressions of other *PAL* gene members (*MePAL2* and *MePAL5*) were also suppressed to some extent. Similarily, some studies also speculated that manipulation the expression of single gene member would also affect the rest of family gene members. There are seven *Rac* genes in rice (*O. sativa*), by using highly conserved regions of the two members (*OsRac1* and *OsRac5*) of the whole *OsRac* gene family, Miki et al. (2005) established transgenic lines by specifically RNA silencing of these two genes, meanwhile, suppression of all members of the gene family with variable efficiencies were also observed. Similarily, silencing *GhOPR9(12-oxo-phytodienoic acid reductases)* gene in cotton (*Gossypium hirsutum*) also cause the suppression of *GhOPR3* expression (Liu et al., 2020). In plants, RNAi- and VIGS-based technologies successfully silenced the specific gene members without influencing the transcriptions of the rest of closely related family members or simultaneously silenced a few family members to overcome functional redundancy (Burch-Smith et al., 2004; Hwang and Gelvin, 2004). However, in certain rigid situation, 100 percent of none off-target was inevitable. In present study, the VIGS primers were specifically designed that only target *MePAL6* (**Supplementary Figure S4**), we assumed that off-target effect can be minimize, in fact, the target gene *MePAL6* showed sufficient suppression here. In the other hand, silencing *MePAL6* may also change the expression of some downstream synthesis genes, i.e, *CCR* and *4CL*. This cascade effect of gene expression in specific metabolites biosynthesis pathway is common in several studies. In the *GhENODL6* (*Early nodulin-like protein 6*) silenced cotton, the transcriptions of both *PAL* and *4CL* genes significantly decreased, resulting in the reduction of SA content (Zhang et al., 2022). In addition, in the *GhOPR9* silenced cotton, expression alterations of other jasmonic acid pathway related genes, like *lipoxygenase* (*LOX*), *allene oxide cyclase* (*AOC*) and *allene oxide synthase* (*AOS*), were also measured. In present study, silencing of *MePAL6* cause systematic impact on the gene expression of lignin biosynthesis pathway, which resulted in decrease of lignin content. The gene expression and metabolite changes lead to the deterioration of TSSM-resistance, as the cassava cultivar SC9 would shift from TSSM-resistant plant into a TSSM-susceptible one. In addition, *PAL* genes not only participate in lignin biosynthesis, but also involved in biosynthesis of several important plant defense activators, i.e., salicylic acid, jasmonic acid and ethylene in several plant species (Kim and Hwang, 2014; Jiang and Yan, 2018; Fujimoto et al., 2015). Manipulating the expression of *PAL* gene might simultaneously affect other defensive responses. Thus, we assumed that apart from regulating lignin accumulation, *MePAL6* would probably attribute to several other secondary metabolite-related defensive pathways, which collectively shaped cassava resistance to TSSM.

However, more delicate efforts are still needed to fill the knowledge gap of *MePAL* function in cassava. The *PAL* gene is associates with abiotic and biotic stress such as pathogen infection, mechanical damage, ultraviolet radiation, chemical treatment and extreme temperatures. This study is the first attempt to know about *MePAL6* involved in insect pest resistance. However, gene functions of other *MePALs* have not been sufficiently illustrated. To fully demonstrated how every single *MePAL* gene work independently or collaboratively in specific biological process, will largely benefit the molecular design breeding of novel cassava varieties that are adaptive to abiotic and biotic stress, which can effectively reduce the adverse environmental impacts and resulting cost, ultimately, promoting the cassava production.

## 5 Conclusion

**Figure 7:**
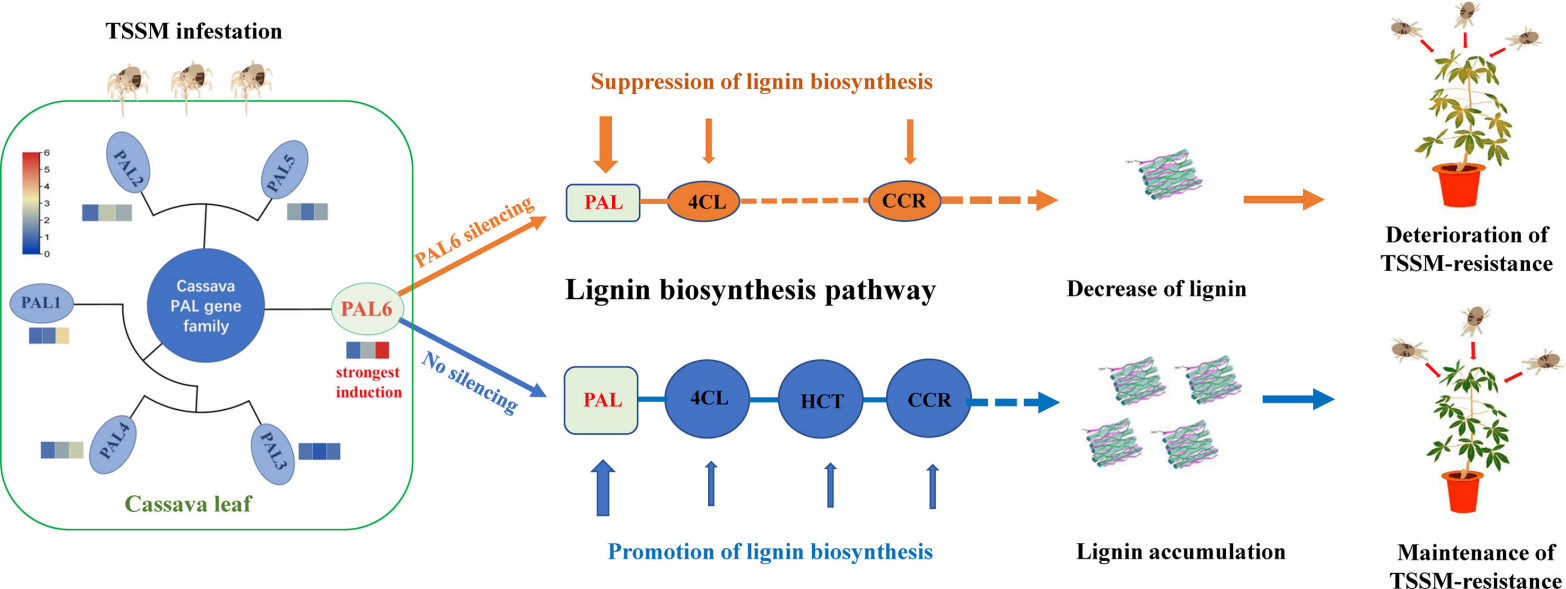
Potential mechanism of *MePAL6* regulates lignin accumulation and shapes cassava resistance to two-spotted spider mite.

A genome-wide analysis of *MePAL* gene family in cassava had identified a total of 6 *MePAL* gene members. Phylogeny, gene duplication and cis-elements analysis implied the potential function of *MePALs* in lignin accumulation as well as insect-pest defense. Silencing of the most strongly induced gene *MePAL6* resulted in suppression of lignin biosynthesis and deterioration of cassava resistance to TSSM, the possible mechanism mentioned above was depicted in **Figure 7**. This study demonstrates the importance role of *PAL* gene and lignin in plants for defending piercing sucking herbivores, and provides insights into potential genes for the molecular breeding of pest-resistant cassava plants.

## Footnotes

1. https://phytozome.jgi.doe.gov/

2. http://pfam.sanger.ac.uk/

3. https://www.ncbi.nlm.nih.gov/

4. https://www.expasy.org/

5. http://meme-suite.org/tools/meme

6. http://bioinformatics.psb.ugent.be/webtools/plantcare/html/

7. https://vigs.solgenomics.net/

8. https://wolfpsort.hgc.jp/

## Data availability statement

The original contributions presented in the study are included in the article/**Supplementary Material**. Further inquiries can be directed to the corresponding author/s.

## Author contributions

XY, XL and QC planned and designed research and experiments; YL, CW, MW, JS, YQ, YZ and YG performed laboratory experiments and analyzed data; XY, XL, QC wrote and edited the paper. XL and QC acquired the funds. All authors contributed to the article and approved the submitted version.
