# Supplementary material for: MePAL6 regulates lignin accumulation to shape cassava resistance against two-spotted spider mite
Source: Front Plant Sci. 2023 Jan 6;13:1067695. doi: 10.3389/fpls.2022.1067695 (PMC9853075; doi:10.3389/fpls.2022.1067695)
Supplement: Supplementary file 1 [file DataSheet_1.docx]

**Supplementary Materials**


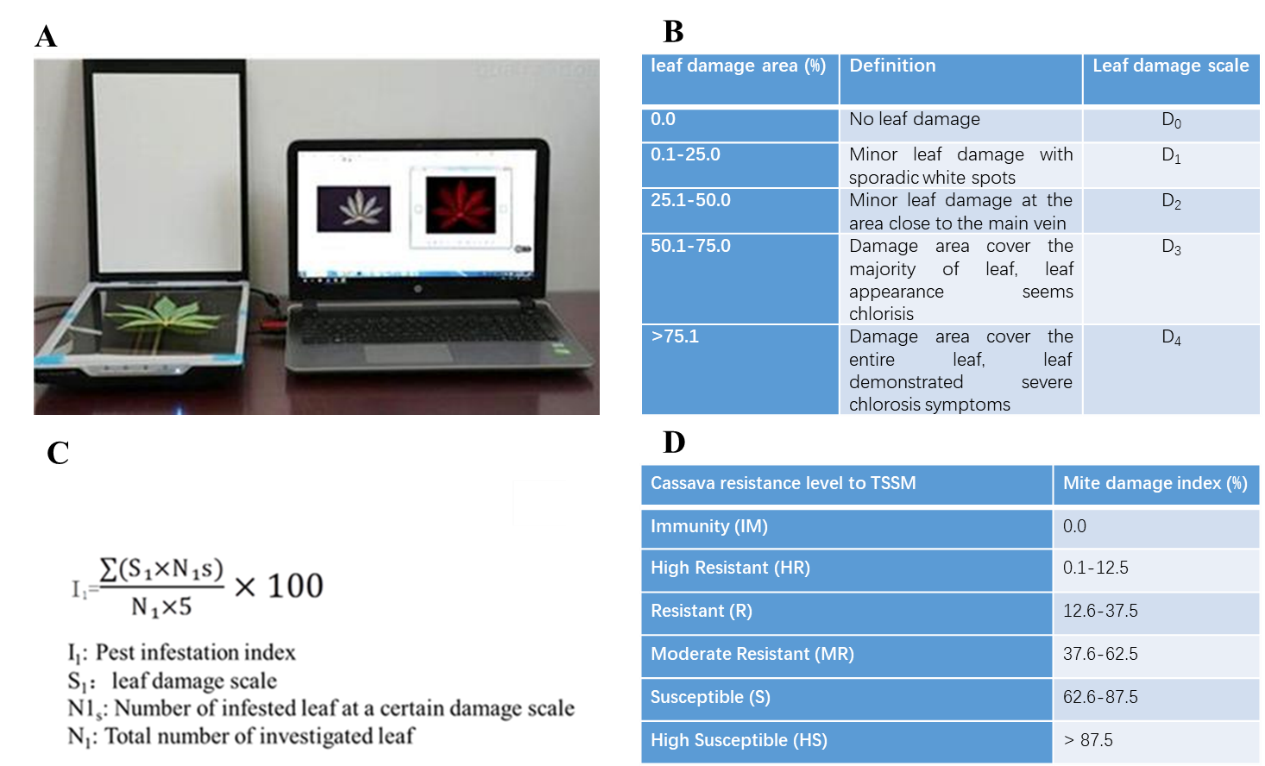


**Figure S1** The methodology of the identification of cassava resistance to TSSM. (A) The leaf damage rate of the cassava plant was analyzed using the Leaf Image Analyzer (YMJ-E, Daji Co. Ltd, Hangzhou, China); (B) Percentages of leaf damage area (Mean ± SD) and its relevant damage scale of cassava leaves; (C) The formulation to calculate mite damage index (MDI); (D) TSSM infestation refers to the identified resistance level of cassava plants.

**Figure S2** Amino acid sequences alignment of *MePAL* family genes in cassava.


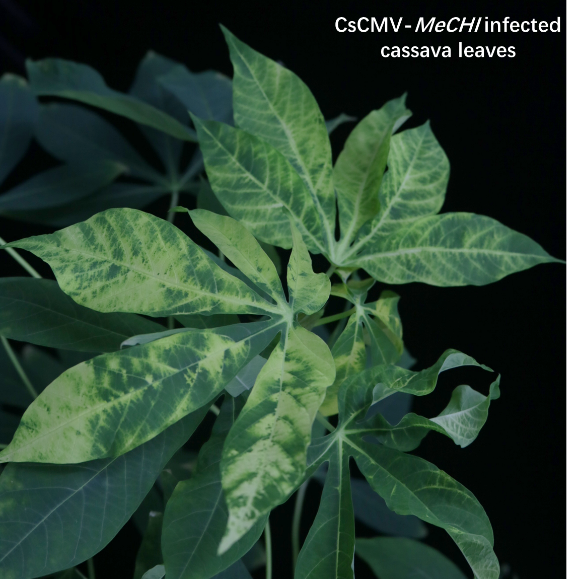


**Figure S3** Phenotypes in cassava cultivar SC9 when applied with VIGS assay at 30 dpi. The plant was infected with CsCMV-*MeCHI* as positive control, and the whitening phenotype indicates gene silencing works.


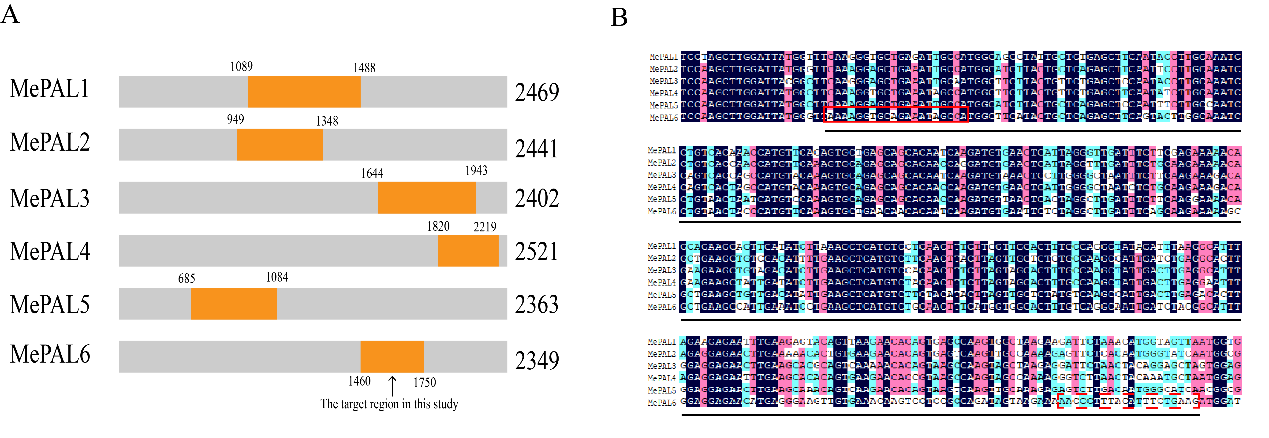


**Figure S4** (A) The optimal target regions (marked in orange) of each *MePAL* gene as analyzed by SGN-VIGS online tool; (B) The target region sequence, the forward and reverse VIGS primer of MePAL6 gene were indicated with black line, red box and red dash box, respectively.

**Table S1** Primers used for different purposes in this study

| Primer name | Primer sequence | Application |
| --- | --- | --- |
| *Meactin-F* | TGATGAGTCTGGTCCATCCA | qRT-PCR |
| *Meactin-R* | CCTCCTACGACCCAATCTCA |  |
| *MePAL*-F | AGGCATTTGGAGGAGAACTTGA | qRT-PCR |
| *MePAL*-R | TCGCAGAATCTTGATGGGTGA |  |
| *MeC4H*-F | GCTCGCCAACAATCCTGCTC | qRT-PCR |
| *MeC4H*-R | CTCCTCCTTCCAACTCCAAACG |  |
| *Me4CL*-R | TCTTCGTCAACGCTTGGAGTA | qRT-PCR |
| *Me4CL*-R | TCTTCGTCAACGCTTGGAGTA |  |
| *MeCCR*-F | GTCAATGCTGTTCAAGGCTATGTG | qRT-PCR |
| *MeCCR*-R | AAATGTATCGCCCGGAGGC |  |
| *MeHCT*-F | GACCTTGTGGTGCCGAGATTC | qRT-PCR |
| *MeHCT*-R | CGTCCAGCCATCGGATAGAAC |  |
| *MeCSE*-F | TCCAATCGGAACCCAACACG | qRT-PCR |
| *MeCSE*-R | AAGAGCAGCCCATACACGAAGAG |  |
| *MeCOMT*-F | GGCTGACCACTCAACCATTACC | qRT-PCR |
| *MeCOMT*-R | GACCGCTCCAGTTCCACCA |  |
| *MeCCoAOMT*-F | GCCCACATCAATGGCTTCC | qRT-PCR |
| *MeCCoAOMT*-R | GCTCCTTCATAGGTTCAGGCTCT |  |
| *MeF5H*-F | GGTGGCATCGGCAATAGAGTG | qRT-PCR |
| *MeF5H*-R | TGCAAGAGGAGTGGGATAGGTG |  |
| *MeCAD*-F | ACCAGAGGGAATGTCACCAGAA | qRT-PCR |
| *MeCAD*-R | CCCTACTCCTCCAAGCCCTAAT |  |
| *MePAL1*-F | ACTCCTAACTGGCAGACC | qRT-PCR |
| *MePAL1*-R | AACAATAGCAAGACCCTC |  |
| *MePAL2*-F | TGAGACCCTGACCGTAGC | qRT-PCR |
| *MePAL2*-R | CAAAGCCAGTGGTGATGC |  |
| *MePAL3*-F | TTGCTCAAAGTGGTGGAT | qRT-PCR |
| *MePAL3*-R | GTGCTGGCATTCTTCTCA |  |
| *MePAL4*-F | GACCTCGTGCCATTATCT | qRT-PCR |
| *MePAL4*-R | AGCCTTCCTTCGCTTGTA |  |
| *MePAL5*-F | GAAGCCTAAACAAGACAGA | qRT-PCR |
| *MePAL5*-R | TTCCTGGAGACATCAATC |  |
| *MePAL6*-F | AGGCATTTGGAGGAGAACTTGA | qRT-PCR |
| *MePAL6*-R | TCGCAGAATCTTGATGGGTGA |  |
| CsCMV-F | TGGGCGCTAATTAGTTTACTGCA | VIGS |
| CsCMV-R | GGTCAAGACGGCTCAACTCTTCA |  |
| CsCMV-*MePAL6*-F | AGTGGTCTCTGTCCAGTCCTAAAAGGTGCAGAAATAGC | VIGS |
| CsCMV-*MePAL6*-R | GGTCTCAGCAGACCACAAGTCTTCAGAAATGTAAAGGGTT |  |

**Table S2** Physicochemical characteristics of cassava *MePAL* gene family members

| Gene | Gene ID | Length (aa) | pI | Mw (Da) | Sublocation |
| --- | --- | --- | --- | --- | --- |
| *MePAL1* | Manes.04G018000 | 588 | 5.93 | 64474 | Endoplasmic reticulum |
| *MePAL2* | Manes.07G098700 | 703 | 6 | 76969.84 | Chloroplast |
| *MePAL3* | Manes.08G008400 | 712 | 6.31 | 77430.29 | Chloroplast |
| *MePAL4* | Manes.09G063700 | 790 | 6.19 | 86144.45 | Chloroplast |
| *MePAL5* | Manes.10G047500 | 710 | 6.03 | 77593.56 | Chloroplast |
| *MePAL6* | Manes.16G098200 | 711 | 5.98 | 78157.51 | Chloroplast |

**Table S3** Prediction of cis-acting elements of MePAL family genes

| **ID** | **Start** | **End** | **Cis-element** |
| --- | --- | --- | --- |
| MePAL1 | 1049 | 1099 | CMA3 |
| MePAL1 | 783 | 834 | 60K protein binding site |
| MePAL1 | 440 | 486 | light responsive element |
| MePAL1 | 470 | 516 | light responsive element |
| MePAL1 | 597 | 643 | light responsive element |
| MePAL1 | 682 | 782 | light responsive element |
| MePAL1 | 735 | 781 | light responsive element |
| MePAL1 | 840 | 886 | light responsive element |
| MePAL1 | 888 | 934 | light responsive element |
| MePAL1 | 1105 | 1131 | light responsive element |
| MePAL1 | 1213 | 1239 | light responsive element |
| MePAL1 | 1217 | 1243 | light responsive element |
| MePAL1 | 1497 | 1525 | light responsive element |
| MePAL1 | 1600 | 1626 | light responsive element |
| MePAL1 | 1614 | 1640 | light responsive element |
| MePAL1 | 133 | 178 | MeJA-responsiveness |
| MePAL1 | 1697 | 1742 | MeJA-responsiveness |
| MePAL1 | 1829 | 1876 | gibberellin-responsive element |
| MePAL1 | 609 | 656 | anaerobic induction |
| MePAL1 | 1811 | 1839.5 | cell cycle regulation |
| MePAL1 | 254 | 300 | light responsive element |
| MePAL1 | 643 | 689 | light responsive element |
| MePAL1 | 1748 | 1794 | light responsive element |
| MePAL1 | 1702 | 1748 | light responsive element |
| MePAL1 | 16 | 62 | MYB binding site involved in drought-inducibility |
| MePAL1 | 347 | 393 | MYB |
| MePAL1 | 1096 | 1240 | MYB |
| MePAL1 | 247 | 293 | MYB |
| MePAL1 | 1766 | 1812 | MYB |
| MePAL1 | 1943 | 1989 | MYB |
| MePAL4 | 1925 | 1954 | element for maximal elicitor-mediated activation (2copies) |
| MePAL4 | 1498 | 1545 | abscisic acid responsiveness |
| MePAL4 | 221 | 267 | anaerobic induction |
| MePAL4 | 1175 | 1221 | anaerobic induction |
| MePAL4 | 140 | 186 | light responsive element |
| MePAL4 | 345 | 391 | light responsive element |
| MePAL4 | 660 | 706 | light responsive element |
| MePAL4 | 674 | 720 | light responsive element |
| MePAL4 | 1288 | 1376 | light responsive element |
| MePAL4 | 1404 | 1476 | light responsive element |
| MePAL4 | 1758 | 1759 | light responsive element |
| MePAL4 | 667 | 718 | 60K protein binding site |
| MePAL4 | 1725 | 1776 | 60K protein binding site |
| MePAL4 | 942 | 969 | light responsive element |
| MePAL4 | 966 | 1013 | gibberellin-responsive element |
| MePAL4 | 270 | 316 | MYB |
| MePAL4 | 1823 | 1869 | MYB |
| MePAL2 | 715 | 743 | light responsive element |
| MePAL2 | 99 | 125 | low-temperature responsiveness |
| MePAL2 | 647 | 694 | MYB binding site involved in light responsiveness |
| MePAL2 | 1491 | 1540 | salicylic acid responsiveness |
| MePAL2 | 560 | 606 | light responsive element |
| MePAL2 | 583 | 629 | light responsive element |
| MePAL2 | 1743 | 1789 | light responsive element |
| MePAL2 | 396 | 441 | MeJA-responsiveness |
| MePAL2 | 1477 | 1522 | MeJA-responsiveness |
| MePAL2 | 1408 | 1457 | wound-responsive element |
| MePAL2 | 1928 | 1977 | wound-responsive element |
| MePAL2 | 447 | 500 | light responsive element |
| MePAL2 | 1505 | 1558 | light responsive element |
| MePAL2 | 583 | 629 | abscisic acid responsiveness |
| MePAL2 | 1738 | 1795 | abscisic acid responsiveness |
| MePAL2 | 912 | 965 | light responsive element |
| MePAL2 | 1786 | 1835 | cell cycle regulation |
| MePAL2 | 460 | 506 | anaerobic induction |
| MePAL2 | 660 | 705 | anaerobic induction |
| MePAL2 | 740 | 786 | anaerobic induction |
| MePAL2 | 115 | 181 | light responsive element |
| MePAL2 | 196 | 242 | light responsive element |
| MePAL2 | 343 | 389 | light responsive element |
| MePAL2 | 880 | 968 | light responsive element |
| MePAL2 | 1008 | 1052 | light responsive element |
| MePAL2 | 1098 | 1134 | light responsive element |
| MePAL2 | 1102 | 1148 | light responsive element |
| MePAL2 | 1331 | 1377 | light responsive element |
| MePAL2 | 1556 | 1602 | light responsive element |
| MePAL2 | 391 | 436 | MeJA-responsiveness |
| MePAL2 | 1476 | 1522 | MeJA-responsiveness |
| MePAL2 | 1788 | 1834 | MYBHv1 binding site |
| MePAL2 | 353 | 402 | defense and stress responsiveness |
| MePAL2 | 463 | 499 | MYB |
| MePAL2 | 1423 | 1479 | MYB |
| MePAL2 | 1745 | 1791 | MYB |
| MePAL2 | 1885 | 1931 | MYB |
| MePAL2 | 599 | 645 | MYB |
| MePAL2 | 29 | 75 | meristem expression |
| MePAL3 | 510 | 557 | MYB binding site involved in light responsiveness |
| MePAL3 | 217 | 266 | salicylic acid responsiveness |
| MePAL3 | 708 | 734 | light responsive element |
| MePAL3 | 736 | 783 | light responsive element |
| MePAL3 | 1272 | 1319 | light responsive element |
| MePAL3 | 470 | 519 | light responsive element |
| MePAL3 | 1922 | 1970 | light responsive element |
| MePAL3 | 908 | 961 | light responsive element |
| MePAL3 | 1617 | 1663 | MYB binding site involved in drought-inducibility |
| MePAL3 | 524 | 570 | light responsive element |
| MePAL3 | 289 | 338 | wound-responsive element |
| MePAL3 | 1607 | 1653 | anaerobic induction |
| MePAL3 | 1903 | 1953 | light responsive element |
| MePAL3 | 478 | 528 | light responsive element |
| MePAL3 | 1774 | 1823 | light responsive element |
| MePAL3 | 147 | 193 | light responsive element |
| MePAL3 | 204 | 250 | light responsive element |
| MePAL3 | 455 | 501 | light responsive element |
| MePAL3 | 642 | 695 | light responsive element |
| MePAL3 | 819 | 865 | light responsive element |
| MePAL3 | 1305 | 1351 | light responsive element |
| MePAL3 | 1738 | 1784 | light responsive element |
| MePAL3 | 520 | 569 | defense and stress responsiveness |
| MePAL3 | 27 | 73 | meristem expression |
| MePAL3 | 1769 | 1816 | light responsive element |
| MePAL3 | 439 | 484 | MYB |
| MePAL3 | 729 | 775 | MYB |
| MePAL3 | 1634 | 1674 | MYB |
| MePAL3 | 1753 | 1793 | MYB |
| MePAL5 | 1813 | 1859 | MYBHv1 binding site |
| MePAL5 | 983 | 1033 | 60K protein binding site |
| MePAL5 | 1724 | 1774 | light responsive element |
| MePAL5 | 1352 | 1401 | light responsive element |
| MePAL5 | 1583 | 1629 | meristem expression |
| MePAL5 | 1899 | 1945 | meristem expression |
| MePAL5 | 1217 | 1263 | light responsive element |
| MePAL5 | 1768 | 1814 | light responsive element |
| MePAL5 | 1217 | 1263 | light responsive element |
| MePAL5 | 1763 | 1814 | light responsive element |
| MePAL5 | 428 | 474 | MYB binding site involved in drought-inducibility |
| MePAL5 | 713 | 761 | light responsive element |
| MePAL5 | 1498 | 1544 | low-temperature responsiveness |
| MePAL5 | 903 | 952 | salicylic acid responsiveness |
| MePAL5 | 954 | 1027 | light responsive element |
| MePAL5 | 1878 | 1944 | anaerobic induction |
| MePAL5 | 1865 | 1931 | anaerobic induction |
| MePAL5 | 1593 | 1646 | light responsive element |
| MePAL5 | 1673 | 1716 | light responsive element |
| MePAL5 | 1172 | 1220 | light responsive element |
| MePAL5 | 1221 | 1270 | abscisic acid responsiveness |
| MePAL5 | 1763 | 1820 | abscisic acid responsiveness |
| MePAL5 | 12 | 65 | light responsive element |
| MePAL5 | 151 | 204 | light responsive element |
| MePAL5 | 791 | 844 | light responsive element |
| MePAL5 | 1026 | 1069 | light responsive element |
| MePAL5 | 230 | 278 | light responsive element |
| MePAL5 | 1602 | 1648 | MYB |
| MePAL5 | 1678 | 1724 | MYB |
| MePAL5 | 1882 | 1928 | MYB |
| MePAL6 | 87 | 136 | wound-responsive element |
| MePAL6 | 1626 | 1674 | light responsive element |
| MePAL6 | 1842 | 1887 | MeJA-responsiveness |
| MePAL6 | 1712 | 1757 | abscisic acid responsiveness |
| MePAL6 | 355 | 401 | anaerobic induction |
| MePAL6 | 997 | 1046 | light responsive element |
| MePAL6 | 1717 | 1763 | light responsive element |
| MePAL6 | 832 | 880 | light responsive element |
| MePAL6 | 485 | 531 | light responsive element |
| MePAL6 | 534 | 601 | light responsive element |
| MePAL6 | 872 | 918 | light responsive element |
| MePAL6 | 943 | 993 | light responsive element |
| MePAL6 | 453 | 501 | light responsive element |
| MePAL6 | 1837 | 1882 | MeJA-responsiveness |
| MePAL6 | 1700 | 1746 | MYB |
| MePAL6 | 242 | 288 | MYB |
| MePAL6 | 1345 | 1391 | MYB |
| MePAL6 | 1700 | 1746 | MYB |
| MePAL6 | 1345 | 1391 | MYB |
| MePAL6 | 1700 | 1746 | MYB |

**Table S4** *MePAL* gene pairs and Ka/Ks values

| **Block code** | **Homologous gene pair** | **Ka/Ks** |
| --- | --- | --- |
| 1 | MePAL1-MePAL2 | 0.071895792 |
| 2 | MePAL1- MePAL3 | 0.066126344 |
| 3 | MePAL1-MePAL4 | 0.089448263 |
| 4 | MePAL1-MePAL5 | 0.072812374 |
| 5 | MePAL2-MePAL3 | 0.069315227 |
| 6 | MePAL2-MePAL4 | 0.08122828 |
| 7 | MePAL2-MePAL5 | 0.095167379 |
| 8 | MePAL3-MePAL4 | 0.077107372 |

Ka/Ks≫1 means positive selection, Ka/Ks≪1 means purifying selection and Ka/Ks = 1means neutral evolution.
